# Quantitative Evaluation of the Sarcomere Network of Human hiPSC-Derived Cardiomyocytes Using Single-Molecule Localization Microscopy

**DOI:** 10.3390/ijms21082819

**Published:** 2020-04-17

**Authors:** Heiko Lemcke, Anna Skorska, Cajetan Immanuel Lang, Lisa Johann, Robert David

**Affiliations:** 1 Department of Cardiac Surgery, Reference and Translation Center for Cardiac Stem Cell Therapy (RTC), Rostock University Medical Center, 18057 Rostock, Germany; Heiko.Lemcke@med.uni-rostock.de (H.L.); anna.skorska@med.uni-rostock.de (A.S.); lisa.johann@uni-rostock.de (L.J.); 2 Faculty of Interdisciplinary Research, Department Life, Light & Matter, University Rostock, 18059 Rostock, Germany; 3 Department of Cardiology, Rostock University Medical Center, 18057 Rostock, Germany; cajetan.lang@med.uni-rostock.de

**Keywords:** human induced pluripotent stem cells, super resolution, maturation, sarcomere network, photoactivated localization microscopy, cardiomyocyte

## Abstract

The maturation of iPSC-derived cardiomyocytes is still a critical point for their application in cardiovascular research as well as for their clinical use. Although multiple differentiation protocols have been established, researchers failed to generate fully mature cardiomyocytes in vitro possessing identical phenotype-related and functional properties as their native adult counterparts. Besides electrophysiological and metabolic changes, the establishment of a well structured sarcomere network is important for the development of a mature cardiac phenotype. Here, we present a super resolution-based approach to quantitatively evaluate the structural maturation of iPSC-derived cardiomyocytes. Fluorescence labelling of the α-actinin cytoskeleton and subsequent visualization by photoactivated localization microscopy allows the acquisition of highly resolved images for measuring sarcomere length and z-disc thickness. Our image analysis revealed that iPSC and neonatal cardiomyocyte share high similarity with respect to their sarcomere organization, however, contraction capacity was inferior in iPSC-derived cardiac cells, indicating an early maturation level. Moreover, we demonstrate that this imaging approach can be used as a tool to monitor cardiomyocyte integrity, helping to optimize iPSC differentiation as well as somatic cell direct-reprogramming strategies.

## 1. Introduction

In recent years, induced pluripotent stem cell (iPSC) technology has become a major technology in cardiovascular research as it enables efficient in vitro generation of functional cardiomyocytes (CM) without any ethical concerns. CM derived from iPSCs have been shown to be suitable for regenerative therapies and drug development [1,2,3]. However, the proper maturation is a common problem as iPSC-CM do not display the same morphological and functional features as their adult counterparts, rather resembling fetal CM [4,5]. In this regard, it has been found that the low level of maturation can be critical in drug development since immature CM seem to be more susceptible to HERG blockers than mature CM [6]. Hence, there is a need for methods that allow monitoring of cardiac maturity in order to improve culture conditions that facilitate proper cardiac development. Moreover, these techniques can help to control the efficiency of stem cell and somatic cell (re-)programming strategies aiming towards the generation of cardiomyocytes from various cell types [7,8,9]

The maturation process of iPSC CM involves several developmental changes of cellular function and physiology, including metabolic switch, electrophysiological and molecular transformation, and changes of mechanical behavior [10,11]. Moreover, structural organization of the contractile machinery is critical during cardiac maturation as it mediates force generation and cell contraction [1]. While adult CM are characterized by a well aligned, highly organized sarcomere network, iPSC-derived CM commonly demonstrate disarranged sarcomeric filaments, thus possessing a lower contraction capability which is more comparable to fetal, immature cardiac cells [12,13].

To evaluate the structural maturation of iPSC CMs, cells are usually subjected to microscopic approaches that allow the quantification of sarcomere organization and alignment [14,15,16]. While electron microscopy provides high spatial resolution, sample preparation and image acquisition is complex and extensive user skills are required. Therefore, fluorescence microscopy is commonly applied to analyze the contractile apparatus in cardiac cells. However, the resolution limit of conventional microscopy is 200–250 nm which makes it difficult to detect and measure sarcomere organization in detail. In contrast to conventional fluorescence imaging, the resolution of photoactivated localization microscopy (PALM) is approximately 10 times higher and allows a more accurate analysis, detecting even subtle alterations of cellular structures [17]. We have applied PALM for image acquisition of the α-actinin labelled sarcomere network of iPSC-derived CM. Subsequent image analyses revealed that this approach is suitable to quantitatively measure sarcomere length and z-disc thickness, two parameters that are importantant in the evaluation of the structural maturity of CMs [18]. Comparison between neonatal cardiac cells and iPSC CM revealed a similar organization of the sarcomere network, while adult CM demonstrated an increased sarcomere length. Correlation with functional data showed that the contraction capacity related to cell size is superior in neonatal cells if compared to iPSC-derived CM, which confirms their immature structural phenotype.

## 2. Results

**Figure 1:**
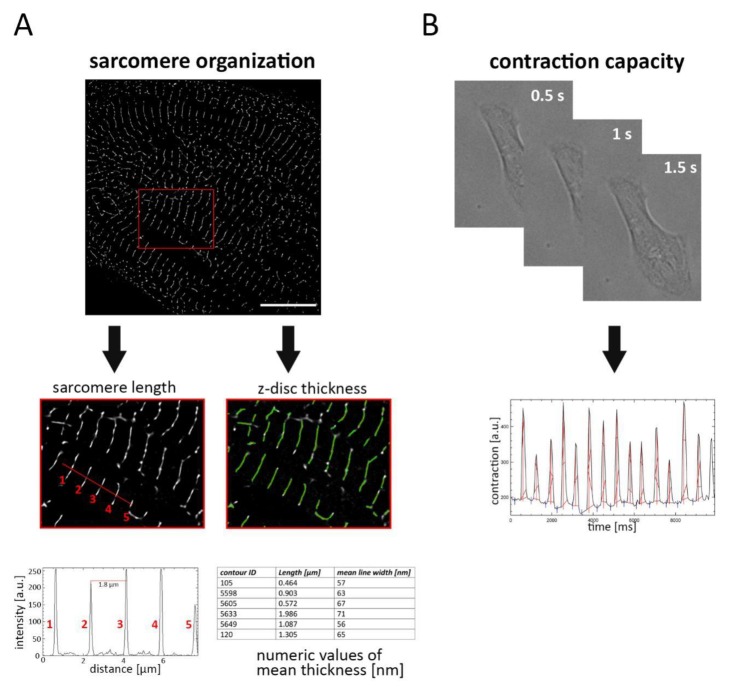
Strategy for investigating the sarcomere organization and cell contraction in cardiomyocytes (CM). (**A**) CM were labelled with α-actinin antibody and subjected to photoactivated localization microscopy (PALM) imaging. Following image acquisition and data reconstruction, sarcomere length and z-disc thickness were determined. Sarcomere length was evaluated by measuring the distance between intensity peaks, corresponding to adjacent α-actinin labelled filaments (1–5). z-disc thickness was automatically analyzed, and the mean width of each filament was calculated. (**B**) To estimate contraction capacity, beating single cells were captured and cell movement was estimated by measuring the change of pixel intensity. Scale bar 10 µm.

To quantitatively evaluate the structural maturation of iPSC-derived CM, the sarcomere network was visualized by labelling α-actinin filaments and samples were subjected to PALM imaging (Figure 1). The thickness of labelled filaments (z-disc) was detected using plugin-based image analysis software that automatically estimates the mean width of each filament. For determination of sarcomere length, the distance between two intensity peaks was measured, corresponding to adjacent α-actinin structures (Figure 1A).

Proper organization of the sarcomere network is directly associated with cellular contraction. Therefore, we asked whether sarcomere organization influences the beating behavior of cardiac cells, and we assessed cellular contraction by video analysis of a single CM. Based on dynamic changes in pixel intensity between image frames, movement during contraction and relaxation was determined (Figure 1B).

**Figure 2:**
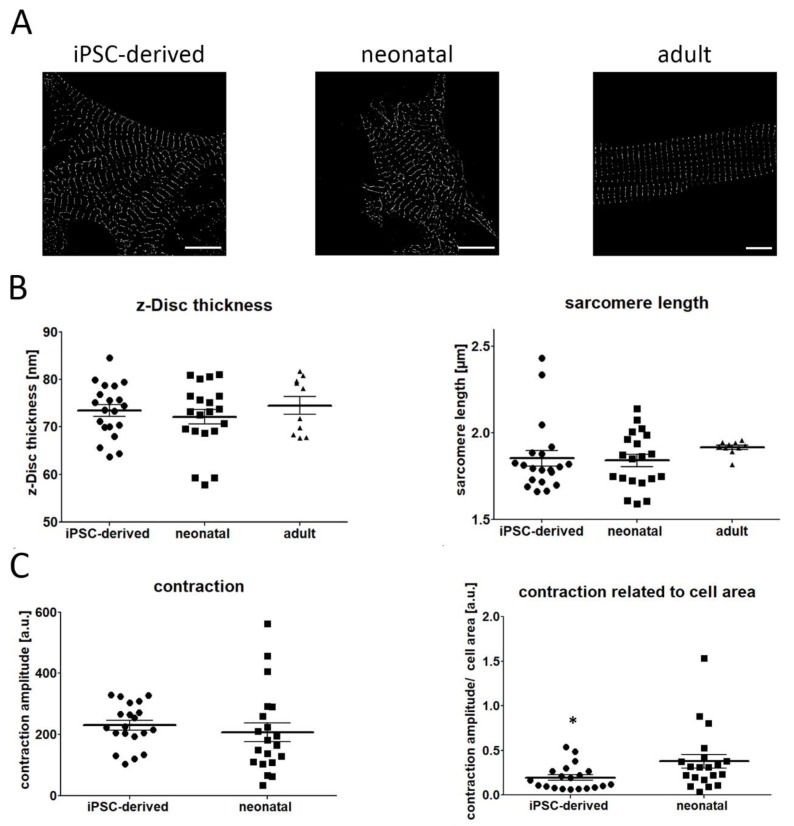
Comparison of sarcomere organization and cell contraction in iPSC-derived, neonatal, and adult CM. (**A**) Representative PALM images of the α-actinin network of investigated cell types. (**B**) Quantitative evaluation showed high similarity in z-disc thickness between iPSC, neonatal and adult CM (iPSCs vs. neonatal vs. adult: 73.45 ± 1.24 vs. 72.12 ± 1.56 vs. 74.6 ± 1.85). Likewise, no difference was detected for sarcomere length in iPSC-derived CM and neonatal cells (iPSCs vs. neonatal: 1.85 ± 0.046 vs. 1.84 ± 0.036), while adult CM demonstrated increased distances between individual sarcomere filaments (1.91 ± 0.01). (**C**) Contraction capacity was found to be similar in both iPSC-derived and neonatal CM. However, if contraction data were normalized to cell size, a reduced beating movement was measured in iPSC CM. Data are presented as mean ± SEM, *n* = 20 iPSC and neonatal CM, *n* = 10 adult CM. Statistical significance was determined using the one-way ANOVA and Student’s *t*-test. * *p* < 0.05. Scale bar 10 µm.

Using this strategy, we compared the sarcomere length and z-disc thickness between iPSC-derived CM and cardiac cells isolated from adult and neonatal heart tissue. Figure 2A demonstrates representative PALM images, showing a similar pattern of α-actinin filaments in both iPSC and neonatal CM. In contrast, adult CM exhibit a much more organized sarcomere pattern. Our quantitative data revealed that the length and thickness of labelled α-actinin structures were almost identical in iPSC and neonatal CM. While the mean thickness of sarcomere structures was about 73 nm, the mean distance between individual sarcomeres was approximately 1.84 µm (Figure 2B), indicating an early, pre-mature developmental stage of iPSC-derived cells. A similar z-Disc thickness was also found in adult CM (~74 nm), whereas sarcomere length was increased (~1.91 µm) if compared to CM obtained from iPSC and neonatal heart tissue.

In a next step we investigated the contractility of iPSCs and neonatal CM by analyzing time-lapse data of beating cells. Contraction amplitude was slightly higher in iPSC CM, although no significant difference was observed (Figure 2C). However, given that larger cells possess increased contraction strength, we calculated the contraction amplitude in relation to the cell area. Following normalization to cell size, we found an increased contraction movement in neonatal cells, if compared with iPSC CM.

**Figure 3:**
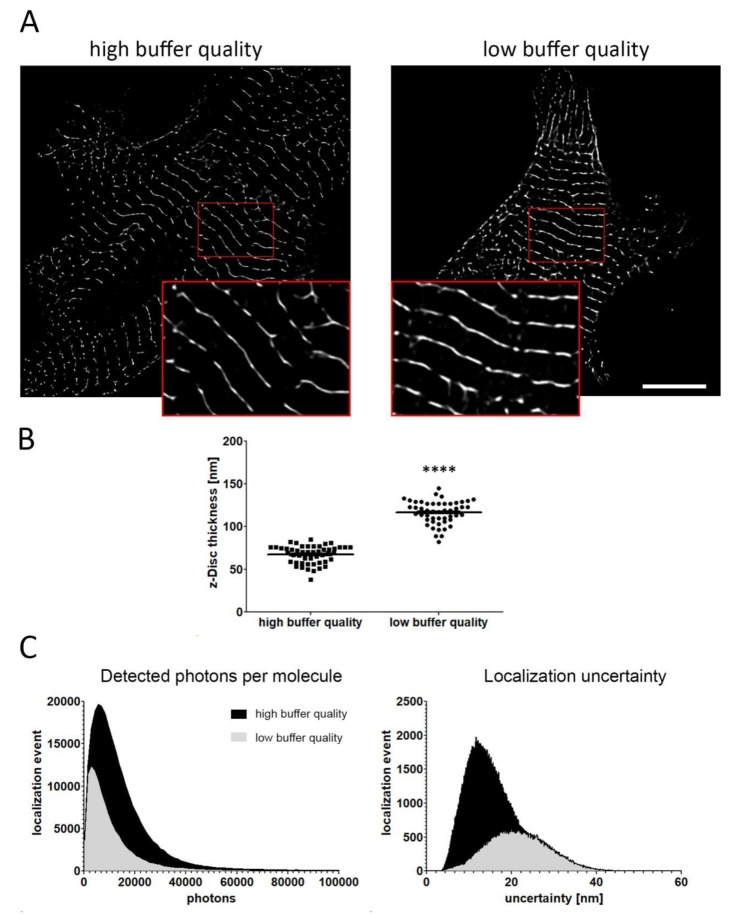
Impact of imaging conditions on data accuracy in PALM. (**A**) Representative images of iPSC-derived CM acquired with freshly prepared and used imaging buffer. Magnified sections (red frame) revealed differences in z-disc thickness when imaging conditions deteriorate. (**B**) Quantitative assessment demonstrated a significant difference in the thickness of sarcomere filaments, showing a much lower z-Disc size when imaging was performed with freshly prepared buffer (fresh vs. old buffer: 67.35 ± 1.05 nm vs. 116.6 ± 1.95 nm). (**C**) Low buffer quality reduces the number of detected photons and strongly impairs localization precision. Data are presented as mean ± SEM. 50 filaments have been subjected to analysis. Statistical significance was determined using the Student’s *t*-test. **** *p* < 0.0001. Scale bar 10 µm.

In contrast to conventional fluorescence microscopy, including confocal imaging, single-molecule localization microscopy dramatically increases the spatial resolution. Therefore, appropriate imaging conditions are critical for the acquisition process as subtle changes can influence the localization precision of detected fluorophores, and thus impair the overall resolution of the final PALM image. For example, the quality of the PALM imaging buffer strongly dictates the photophysical properties of the fluorescent dye. As shown in Figure 3, freshly prepared imaging buffer demonstrates a much better image quality and improved data accuracy when compared to buffer that has been prepared 24 h before imaging. Labelled α-actinin filaments appear thinner when images were acquired under adequate imaging conditions. Quantitative assessment confirmed a profoundly lower z-Disc thickness following microscopy with fresh imaging buffer (fresh vs. old buffer: 67.35 ± 1.05 nm vs. 116.6 ± 1.95 nm) (Figure 3B).

The observed lack of data accuracy is based on an impaired blinking capability of the fluorescent dye, as imaging buffer of inferior quality results in a lower number of detected photons per molecule (Figure 3C). Moreover, localization uncertainty is reduced, which provokes a decrease of the lateral resolution (Figure 3C). These data highlight the importance of appropriate imaging conditions when evaluating sarcomere organization in cardiac cells using single-molecule localization microscopy.

## 3. Discussion

The immature phenotype of iPSC derived CMs limits their potential for cell-based therapies, drug development and cardiovascular research. Although many strategies have been applied to improve the cardiac differentiation and maturation of stem cells, researchers failed to generate iPSCs CMs with properties matching their adult counterparts [4]. In this regard, it is of uttermost importance to provide techniques that help to evaluate the maturation level of iPSC-derived CMs. Here, we present a super resolution-based method to quantitatively analyze sarcomere filaments of individual iPSC-derived CMs and correlate with their contraction capability. Since the organization of the sarcomere directly reflects the structural maturation state, this technique enables monitoring of the cardiac development of iPSCs.

PALM imaging is based on the detection of single fluorescent molecules and offers among the highest resolutions in light microscopy currently available (20–50 nm) [19]. Therefore, PALM allows the detection of very subtle alterations of the α-actinin cytoskeleton in iPSC-derived CM that are barely or not detectable by classical fluorescence microscopy. However, to achieve such high spatial resolution, PALM requires defined imaging conditions, e.g., photophysical properties of the selected fluorophores [20]. Another critical point is the imaging buffer used to promote rapid switching between the fluorescent and dark states of the fluorescent dye. Our results highlight the impact of buffer quality on image acquisition (Figure 3). Blinking efficiency of the fluorophore and localization uncertainty is strongly affected when PALM is performed with imaging buffer of poor quality.

The acquired PALM data indicate that the organization of the sarcomere network in iPSC-derived CM is comparable to neonatal CM as no significant differences have been found for sarcomere length and z-Disc thickness. This is in agreement with numerous studies showing that the differentiation status of CMs generated from stem cells resemble an embryonic or neonatal state [4,21,22]. The average sarcomere length in iPSC-derived CM is about 1.84 µm, which corresponds to former reports showing that the distance between two adjacent sarcomeres is ~1.7–2.0 µm (Figure 2) [14,16,23]. This large variety in sarcomere length, observed in several studies, might be attributed to different imaging techniques used, causing varying extents of artefacts [24,25]. In addition, applied differentiation protocols and the used iPSC cell lines might have an impact on the level of structural maturation.

In contrast to sarcomere length, accurate detection of z-disc thickness by fluorescence imaging is even more challenging as its size is far beyond the classical resolution limit of light microscopy. Using electron microscopy, previous studies demonstrated that the thickness of z-lines in iPSC-derived cardiac cells ranges from 50 nm to 80 nm [15,26,27] which is similar to our results (~73 nm, Figure 2) [15,26,27,28].

The maturation state of the sarcomere network directly correlates with the contraction capacity of cardiac cells. Sheehy and colleagues revealed that the contractile performance in neonatal cells is superior to iPSC-derived CMs [29]. In another study, engineered myocardium from neonatal mouse ventricles demonstrated significantly higher contraction strength than myocardium generated from stem cells [28]. Similarly, we obtained data showing improved cell contraction in neonatal cells when cell size is considered.

Taken together, we have presented an imaging-based strategy to monitor subtle changes in the sarcomere network, determining the structural maturation of CM. As such, this approach can be applied to establish and evaluate strategies to improve the cardiac development of iPSC-derived CM towards a more adult-like phenotype. In addition to the optimization of differentiation protocols, our method further represents a valuable tool to control the efficiency and success of cardiac direct (re)programming using somatic cell types.

## 4. Materials and Methods

### 4.1. iPCS Culture and Cardiac Differentiation

Cell culture of iPSCs and derived cardiomyocytes was performed on Laminin521 (Biolamina, Sundbyberg, Sweden) coated surfaces. iPSCs (Takara Bio Inc, Kusatsu, Japan) were maintained in iPS Brew (Miltenyi Biotec, Bergisch Gladbach, Germany), supplemented with Zellshield (Biochrom, Berlin, Germany). For cardiac differentiation, cells were seeded on 6 well plates in RPMI 1640 Glutamax media (Thermo Fisher, Waltham, MA, USA) containing 1% sodium pyruvate, 200 µM ascorbic acid (all Sigma Aldrich, St. Louis, USA) 1% Zellshield and 2% B27 without insulin (Miltenyi Biotec) and treated with 1 µM Chir99021, 5 ng/mL basic fibroblast growth factor, 5 ng/mL bone morphogenetic protein 4 and 9 ng/mL Activin A (all Miltenyi Biotec) for three days. Subsequently, cells were cultured in RPMI 1640 Glutamax supplemented with B27 with insulin (Miltenyi Biotec) and 5 mM IWP-2 (Tocris, Bristol, UK) was added for seven days. Metabolic selection of cardiomyocytes was performed between days 13 and 16 using RPMI without Glucose (Thermo Fisher), containing 1% Zellshield, 2.3 mM sodium acetate and 100 µM mercaptoethanol (all Sigma Aldrich).

After 25 days, cardiomyocytes were dissociated using a two-compound dissociation kit (Stemcell technologies, Vancouver, Canada) according to the manufacturer’s instructions. Cells were seeded in 8 well chamber slides and cultured for an additional 5 days (Ibidi, Martinsried, Germany).

### 4.2. Isolation and Culture of Neonatal and Adult Cardiomyocytes

Experiments involving neonatal and adult mice were conducted according to the ethical guidelines for animal care of the Rostock University Medical Centre. Isolation of neonatal CMs was performed as described previously [30]. Following enzymatic digestions, cells were seeded on 8 well chamber slides and cultured in DMEM supplemented with 10% FBS (Pan Biotech, Aidenbach, Germany) and 1% Zellshield (Biochrom) on 0.1% gelatin (Sigma Aldrich) coated surfaces.

Adult murine cardiomyocytes were isolated as described elsewhere [31]. Briefly, adult CM were obtained by injection of collagenase 2/4 solution (Sigma Aldrich) into the left ventricle. Digested heart tissue was cut into smaller pieces and gently triturated with a 1 mL pipette. Isolated cells were seeded on collagen-coated cell surfaces and cultured for one day before being subjected to fluorescence labelling.

### 4.3. Immunofluorescence Labelling of the Sarcomere Network

To visualize the sarcomere network of cardiomyocytes, cells were fixed with 2% paraformaldehyde for 15 min, followed by incubation with 0.2% Triton-X 100 (all Sigma Aldrich) for 5 min. Following treatment with 1% bovine serum albumin, fixed cells were labelled with anti-sarcomeric α-actinin (abcam, ab9465) and goat anti-mouse AlexaFlour647 secondary antibody (Thermo Fisher, A-21237).

### 4.4. PALM Imaging

For single-molecule microscopy, cells were kept in an imaging buffer containing 10% Glucose, 10 mM sodium chloride, 50 mM Tris-HCL, catalase, Pyranose oxidase, 100 mM Cysteamine, 2 mM Cyclooctatetraene and 100 mM Mercaptoethanol (all Sigma Aldrich). A total of 5000–10,000 frames were acquired using a Zeiss ELYRA LSM 780 (Zeiss, Oberkochen, Germany) equipped with a 1.57 N.A. 100x oil objective. Image reconstruction and post-processing were conducted with Image J software and the Thunderstorm plugin [18].

### 4.5. Quantitative Evaluation of the Sarcomere Network and Cardiac Contraction

Following reconstruction of the PALM images, the sarcomere network was analyzed using Image J software. Sarcomere length was evaluated by measuring the distance of intensity peaks between adjacent sarcomere filaments. The length of 20 Sarcomeres per cell was determined. Thickness of z-lines was automatically detected with the Image J integrated ridge detection plugin.

For the assessment of cell contraction, CM were seeded on 8-well chamber slides at low density. Videos of beating single cells were acquired for 20 s with a frame rate of 33 fps using Zeiss ELYRA LSM 780 (Zeiss). Cardiac contraction was measured with the Image J Musclemotion plugin [32]. Mean contraction was calculated for each individual cell.

### 4.6. Statistical Analysis

Data are presented as mean ± standard error of the mean (SEM). Statistical significance was calculated using a two-tailed Student’s *t*-test. Comparison of more than two parameters was performed using a one-way ANOVA. Probability levels considered as statistically significant were * *p*  ≤  0.05 and **** *p*  ≤  0.0001. Calculations and graph analysis were composed with GraphPadPrism5 software (GraphPad Prism, Inc., San Diego, CA, USA).

## Abbreviations

CM	cardiomyocytes
iPSC	induced pluripotent stem cell
PALM	photoactivated localization microscopy
